# Hey bHLH Proteins Interact with a FBXO45 Containing SCF Ubiquitin Ligase Complex and Induce Its Translocation into the Nucleus

**DOI:** 10.1371/journal.pone.0130288

**Published:** 2015-06-12

**Authors:** Daniela Salat, Anja Winkler, Henning Urlaub, Manfred Gessler

**Affiliations:** 1 Developmental Biochemistry, Theodor-Boveri-Institute, Biocenter, University of Wuerzburg, Wuerzburg, Germany; 2 Bioanalytical Mass Spectrometry, Max-Planck-Institute of Biophysical Chemistry, and Bioanalytics, Institute for Clinical Chemistry, University Medical Center, Goettingen, Germany; 3 Comprehensive Cancer Center Mainfranken, Wuerzburg University, Wuerzburg, Germany; Feinberg Cardiovascular Research Institute, Northwestern University, UNITED STATES

## Abstract

The Hey protein family, comprising Hey1, Hey2 and HeyL in mammals, conveys Notch signals in many cell types. The helix-loop-helix (HLH) domain as well as the Orange domain, mediate homo- and heterodimerization of these transcription factors. Although distinct interaction partners have been identified so far, their physiological relevance for Hey functions is still largely unclear. Using a tandem affinity purification approach and mass spectrometry analysis we identified members of an ubiquitin E3-ligase complex consisting of FBXO45, PAM and SKP1 as novel Hey1 associated proteins. There is a direct interaction between Hey1 and FBXO45, whereas FBXO45 is needed to mediate indirect Hey1 binding to SKP1. Expression of Hey1 induces translocation of FBXO45 and PAM into the nucleus. Hey1 is a short-lived protein that is degraded by the proteasome, but there is no evidence for FBXO45-dependent ubiquitination of Hey1. On the contrary, Hey1 mediated nuclear translocation of FBXO45 and its associated ubiquitin ligase complex may extend its spectrum to additional nuclear targets triggering their ubiquitination. This suggests a novel mechanism of action for Hey bHLH factors.

## Introduction

The mammalian Hey protein family comprises a small group of highly conserved basic helix-loop-helix (bHLH) transcription factors with three members: Hey1, Hey2 and HeyL. They play essential roles in cardiovascular development [1] but also during epithelial-to-mesenchymal transition [2], neural development [3, 4], myogenesis [5] and bone development [6]. Hey proteins are closely related to the *D*. *melanogaster* hairy and E(spl) proteins. They show high structural similarity with the Hes protein family, especially in the DNA binding basic domain as well as the HLH and Orange domains that mediate homo- and heterodimerization. The latter also function as a protein interaction platform and they might modulate and stabilize Hey dimerization [7]. Hey proteins are further characterized by two conserved peptide motifs—YRPW and TEIGAF—of unknown function at the C-terminus. In contrast to the WRPW motif of Hes proteins, the YRPW peptide is not able to bind to TLE/groucho type co-repressors, but cognate partners have yet to be found [8].

Several dimerization partners of Hey proteins have been identified before (for review see [9, 10]). Generally, Hey proteins act as transcriptional repressors, but depending on the interaction partner there may be distinct differences in Hey mediated transcriptional regulation. While direct repression of target promoters appears to be the primary mode of action [11], there are also reports on transcriptional activation and indirect actions through competition for dimerization partners or complex formation with other DNA binding proteins [12, 13]. However, the exact mechanism how Hey proteins regulate transcription and whether they actually use these different binding partners *in vivo* is still an open question.

To gain closer insight into Hey1 biochemical functions and behavior we sought to isolate novel Hey1 associated proteins using an unbiased screen by tandem affinity purification and mass spectrometry (MS). Amongst others, we identified the F-box protein FBXO45 as a Hey1 co-purified protein. *FBXO45* was originally characterized as an estrogen inducible gene [14]. Orthologs of *FBXO45* are found in humans (*FBXO45*), mice (*Fbxo45*), *C*. *elegans* (*Fsn-1*) and in *D*. *melanogaster* (*Fsn*) [15]. FBXO45 is mainly expressed in the nervous system and is required for proper neuronal development [15, 16]. F-box proteins function as the substrate recognition subunit in SCF (SKP1-Cullin-F-box) E3-ligase complexes that mediate polyubiquitination of proteins [17]. FBXO45 itself can bind to p73 and triggers its proteasome dependent degradation [18]. In contrast to other F-box proteins, FBXO45 functions as a linker protein within an unusual SCF complex containing PAM (Protein Associated with Myc, also known as MYCBP2) and SKP1 (S-phase Kinase associated Protein 1) [16].

PAM belongs to the conserved family of so-called Phr proteins including *D*. *melanogaster* Highwire, *C*. *elegans* Rpm-1 and zebrafish Esrom [19–23]. This family comprises a group of large proteins with E3-ligase activity through a C-terminal RING finger domain that are key factors in neuronal development [24]. PAM interacts with FBXO45 and stabilizes it through its Myc binding domain [16]. The precise function of SKP1 within the FBXO45 complex is currently not known.

Here we describe the identification and characterization of the FBXO45/SKP1/PAM complex as a new Hey1 associated protein complex and show that Hey1 proteins can induce nuclear accumulation of this ubiquitin ligase complex.

## Methods

### Plasmids

The pcDNA3-Flag-FBXO45 full-length and deletion mutant plasmids were kindly provided by G. Melino [18]. The FBXO45 insert was released with XhoI/BamHI (blunted with T4 polymerase) and inserted into XhoI/BglII (blunted) digested pEGFP-C3 to produce peGFP-FBXO45. To generate peGFP-FBXO45ΔF a PCR amplicon from pcDNA3-Flag-FBXO45ΔF (5’-ctagatcttgcgcccgcagcctgg-3’ and 5’-cagagctctcatccgtccaaaggttttccaagg-3’) was inserted into pEGFP-c1 via BglII/SacI digest. To generate peGFP-FBXO45ΔS a BamHI/PmeI fragment from pcDNA3-Flag-FBXO45ΔS was inserted into BamHI/XhoI (blunted) digested pEGFP-C1. The peGFP-PAM plasmids were all derived from pCMV3B-Myc-PAM (kindly provided by V. Ramesh). For peGFP-PAM-N the BamHI/XbaI digested 4500 bp insert was transferred into the corresponding sites of pEGFP-C1. The peGFP-PAM-M plasmid was made by inserting the XbaI/AflII (blunted) digested PAM fragment into SmaI digested pEGFP-C2. For peGFP-PAM-C the BamHI/PmeI digested PAM fragment (5070 bp) was inserted into pEGFP-C1 digested with BamHI/BclI (blunted). For peGFP-Hey1 ΔbHLH-O the PCR amplified cDNA (5’-gactcgagacaacaactacgcatcccagc-3’ & 5’-gcaagcttttagaaagctccgatctctgtcc-3’) was inserted into pEGFP-C1 via XhoI/HindIII. Plasmid inserts were verified by sequencing.

The expression plasmids peGFP-Hey1 and Hey1 deletion mutants, pCS2P-Flag-Hey1 and pCS2P-Flag-Hey1-RK_3_ were described previously [11, 25]. For pmCherry-Hey1 the SacI/SnaBI digested pCS2P-Flag-Hey1 insert was transferred into the SacI/SmaI digested pmCherry-C1. The pmCherry-Hey2 plasmid was made by inserting the BamHI/SacI Hey2 from pCS2P-Flag-Hey2 into the corresponding site of pmCherry-C1. The plasmids pcDNA3-HA-Ubiquitin and pcDNA3-HA-SKP1 were kindly provided by N. Popov.

### Cell culture

The doxycycline (Dox) inducible HEK293 cell lines (293tet-FS-Hey1, etc.) for regulated expression of Flag-Strep (FS)-Hey1, FS-Hey2 and Flag-HeyL were described previously [11]. For Dox regulated FS-Hes1 expression in HEK293 cells we employed the Tol2 transposase system [26] as described for Flag-HeyL [11].

All cell lines were maintained in DMEM (Sigma-Aldrich, Munich, Germany) containing 10% fetal calf serum (PAN Biotech, Aidenbach, Germany), 50 μg/ml streptomycin and 50 U penicillin (PAN Biotech, Aidenbach, Germany) at 37°C under 5% CO_2_.

Transient transfections for immunoprecipitation and immunofluorescence experiments were performed using the polyethylenimine (PEI) transfection reagent (PEI to DNA ratio: 2:1, for 8 h). All transfections were adjusted to equal DNA amounts with empty vector.

### Tandem affinity purification

293tet-FS-Hey1 cells were plated with a density of 3x 10^6^ cells per 15 cm dish and treated with 100 ng/ml Dox for 72 h to induce FS-Hey1 expression. For whole cell lysates cells were rinsed with cold PBS and lysed with 800 μl per dish of TBS buffer (100 mM Tris pH 8.0, 150 mM NaCl) containing 10% glycerol and 1% Triton-X100. For preparation of nuclear extracts the cells were rinsed with cold PBS followed by hypotonic buffer1 (10 mM Tris pH 8.0, 1.5 mM MgCl_2_, 10 mM KCl, 10% glycerol). Then the cells were incubated for 10 min in 3 packed cell volumes hypotonic buffer2 (10 mM Tris pH 8.0, 1.5 mM MgCl_2_, 10 mM KCl, 0.1% Triton-X100, 10% glycerol). The cell suspension was homogenized and centrifuged (720 g, 10 min, 4°C). The nuclei pellet was washed with hypotonic buffer1, resuspended in one volume of low salt buffer (100 mM Tris pH 8.0, 1.5 mM MgCl_2_, 10% glycerol), carefully mixed with two volumes high salt buffer (100 mM Tris pH 8.0, 1.5 mM MgCl_2_, 840 mM KCl, 10% glycerol) and incubated for 30 min. The nuclear lysate was cleared by ultracentrifugation (38500 g, 30 min, 4°C) and diluted with two volumes of dialysis buffer (100 mM Tris pH 8.0, 0.3% Triton-X100, 10% glycerol). All buffers contained the following inhibitors: Complete protease inhibitors (Roche, Mannheim, Germany), 1 mM PMSF, 1 mM EDTA and 20 mM NaF.

Cleared whole cell lysates as well as nuclear extracts were incubated with 400 μl of a 50% slurry anti-Flag M2 affinity gel (A2220, Sigma-Aldrich, Munich, Germany) at 4°C over night. Flag-beads incubated with whole cell lysates were washed 5 times with wash buffer (TBS containing 0.1% Triton-X100). Bound proteins were eluted in 2 ml buffer containing 150 ng/μl Flag peptide (F3290, Sigma-Aldrich, Munich, Germany) for 2 h. The eluate was incubated with 200 μl StrepTactin beads (IBA, Goettingen, Germany) at 4°C over night. The StrepTactin beads were washed 3 times with wash buffer, proteins were eluted with 200 μl wash buffer containing 2.5 mM desthiobiotin (IBA, Goettingen, Germany) and subjected to mass spectrometry analysis. Flag-beads incubated with nuclear extracts were washed 6 times in TBS buffers (100 mM Tris pH 8.0) containing decreasing concentrations of KCl (100–0 mM), increasing concentrations of NaCl (50–150 mM), 0.1% Triton-X100 and 10% glycerol. Bound proteins were eluted 1 ml elution buffer followed by purification with 90 μl StrepTactin beads and elution in 150 μl Strep elution buffer as described above.

### Mass spectrometry

Affinity purified proteins were separated on pre-cast gels (NuPAGE, Invitrogen), stained with Coomassie and entire gel lanes were cut into 23 gel slices. Proteins within individual gel slices were in-gel digested with trypsin, peptides were extracted and analyzed by LC-coupled MS on an orbitrap XL mass spectrometer under standard conditions. Proteins were identified by database search against NCBInr database (taxonomy human) using MASCOT as search engine. Data were annotated using Scaffold 3.0 software.

### Immunoprecipitation assays

HEK293 cell with inducible FS-Hey1, FS-Hey2, Flag-HeyL and FS-Hes1 expression (100 ng/ml Dox for 72 h) or HEK293T cells were used. Whole cell lysates were prepared in TBS buffer (20 mM Tris pH 7.8, 150 mM NaCl) containing 10% Glycerol, 0.3% Triton-X100, Complete protease inhibitors, 1 mM PMSF, 1 mM EDTA and 20 mM NaF. Cleared lysates were incubated with a 50% slurry of anti-Flag M2 affinity gel at 4°C overnight. The beads were washed 6 times with lysis buffer, resuspended in SDS loading buffer (0.1 M Tris pH 6.8, 4% SDS, 0.25% bromophenol blue, 25% glycerol, 10% 2-mercaptoethanol) and subjected to Western blot analysis.

For ubiquitin assays transiently transfected HEK293T cells were lysed in RIPA buffer (20 mM Tris pH 7.8, 150 mM NaCl, 10% Glycerol, 1% Triton-X100, 1% deoxycholic acid, 0.1% SDS, Complete protease inhibitors, 1 mM PMSF, 1 mM EDTA, 20 mM NaF and 20 mM N-ethylmaleimide). The lysates were subjected to immunoprecipitation with a 50% slurry of anti-HA conjugated agarose (A2095, Sigma-Aldrich, Munich, Germany) at 4°C over night and processed as above.

### Western blot

The following primary antibodies were used: mouse anti-Flag M2 antibody (F3165, Sigma-Aldrich, Munich, Germany, 1:2000), mouse anti-HA antibody (H9658, Sigma-Aldrich, Munich, Germany, 1:2000), goat anti-GFP antibody (AA 246, Antibodies online, 1:1000). Secondary antibodies were peroxidase conjugated rabbit anti-goat IgG (Sigma-Aldrich, Munich, Germany, 1:5000) and peroxidase conjugated goat anti-mouse IgG (AP124P, Chemicon/Millipore, 1:5000).

### Immunofluorescence assay

HeLa cells were cultured on glass coverslips in 24-well plates at a density of 3x 10^4^ cells per well. 24 h after transfection the cells were rinsed with PBS, fixed with 4% PFA and permeabilized with 0.1% Triton-X100 in PBS. Fish skin gelatin (0.2% in PBS; Sigma-Aldrich, Munich, Germany) was used for blocking of nonspecific antibody binding sites. Flag-tagged proteins were detected with the anti-Flag M2 antibody (1:400) and a secondary goat anti-mouse Alexa594 antibody (Invitrogen/Molecular Probes, 1:1000). Nuclei were stained with Hoechst33342 (Roth, Karlsruhe, Germany, 1:10000). Cover slides were mounted with Mowiol. Pictures were taken using a Leica AF6000 fluorescence microscope. 80 cells were counted per preparation.

### Cycloheximide stability assay

293tet-FS-Hey1 cells were grown in 6-well plates and Hey1 expression was induced by Dox (100 ng/ml) addition for 72 h. After 48 h the cells were PEI transfected with pcDNA3-Flag-FBXO45 and incubated for additional 24 h. The cells were then treated with cycloheximide (0.1 M) in absence or presence of the proteasome inhibitor MG132 (Sigma-Aldrich, Munich, Germany, 20 μM). SDS lysates were subsequently analyzed by Western blot.

### Luciferase assay

293tet-FS-Hey1 cells were stably transfected with the luciferase reporter pTol2-mHey1(2.9kb)-Luc containing a 2.9 kb fragment of the Hey1 promoter using Tol2-mediated transposition and hygromycin (150 μg/ml) selection. The cells (6x 10^4^) were treated with different amounts of Dox (50 ng/ml and 500 ng/ml) to induce FS-Hey1 expression for 72h in a 24well format. 24 h prior to lysis the cells were transfected with pcDNA-Flag-FBXO45 (100 ng, 200 ng). Luciferase assays were performed as described previously [11].

## Results

### Identification of novel Hey1 interacting proteins

**Fig 1:**
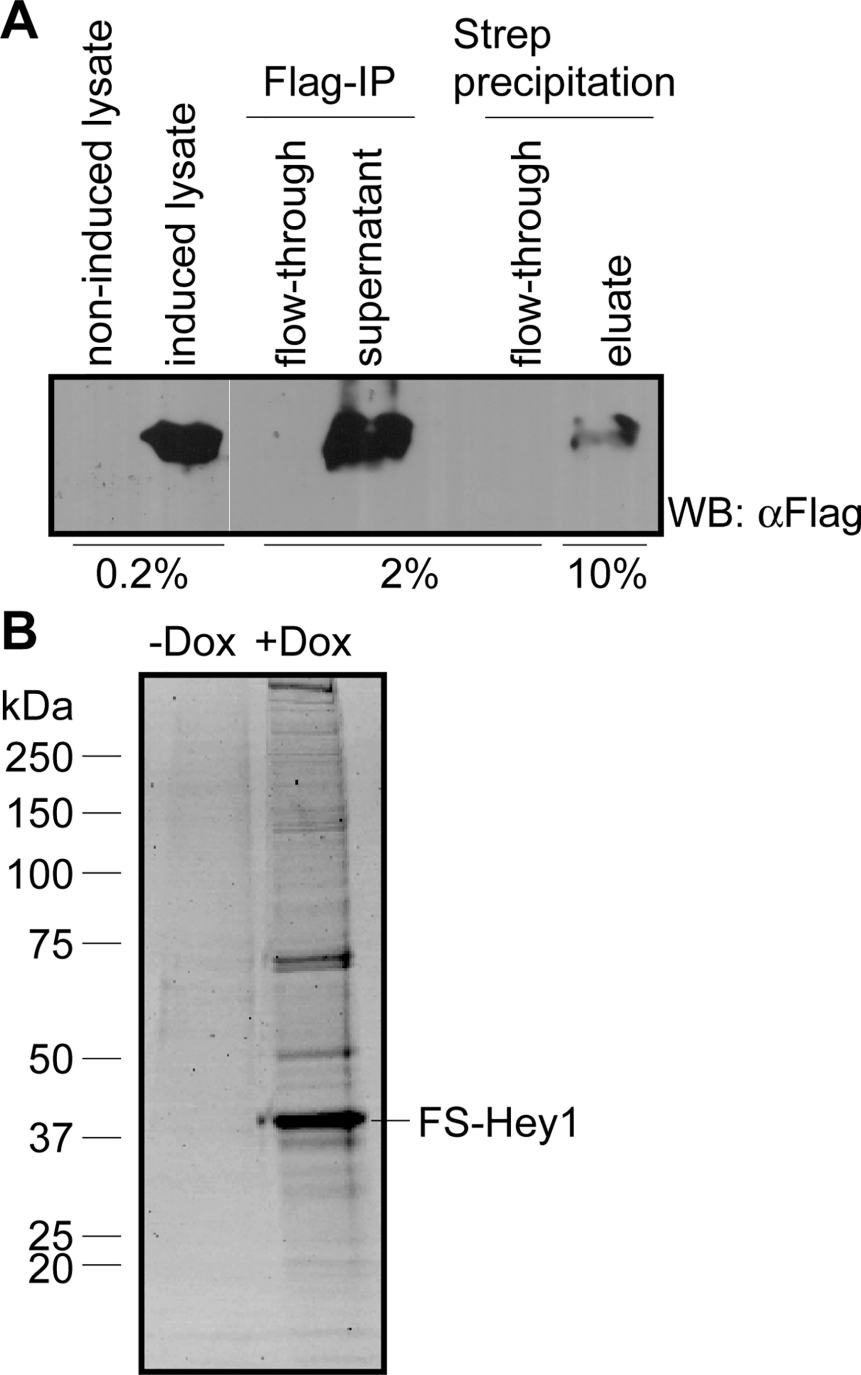
Tandem affinity purification of Hey1 and associated proteins from whole cell lysates. (A) Western blot analysis of individual purification steps from whole cell lysates using an anti-Flag antibody. Numbers correspond to percentage of original lysate loaded. (B) Coomassie staining of tandem purification from whole cell lysates as an example.

In order to identify novel Hey1 interacting proteins we established a tandem affinity purification system for Hey1 containing protein complexes based on the Flag-Strep (FS) tandem tag described by Gloeckner et al. [27]. FS-Hey1 was expressed in stably transduced HEK293-tet cells (293tet-FS-Hey1), where expression can be tightly regulated by Dox [11]. The purification experiments were performed with low levels of recombinant Hey1 expression using 100 ng/ml Dox induction for 72 h. To distinguish putative binding partners from unspecific interactors we used non-induced cells as negative controls. Since Hey proteins localize to the nucleus, we performed co-purifications from whole cell lysates (Fig 1A and 1B) and from nuclear extracts that were expected to yield lower levels of non-specific contaminants. The efficiency of tandem purification was controlled by Western blot using a Flag-tag specific antibody (Fig 1A). Purified complexes were separated by SDS-PAGE followed by Coomassie staining (Fig 1B). Gel lanes were cut into 23 slices and after tryptic digest protein fragments were analyzed by mass spectrometry. In total, 648 co-purified unique proteins were identified from whole cell lysates and 220 unique proteins from nuclear lysates. We focused on proteins that were specifically identified in both co-purification experiments (S1 Table). Hes1, a known Hey1 interaction partner [28], was only purified from cells treated with Dox indicating effective co-purification of Hey1 protein complex partners from induced cells. Members of a previously described E3-ligase complex consisting of the F-box protein FBXO45, the potential E3-ligase PAM as well as the linker protein SKP1 [16] were co-purified repeatedly with high protein coverage percentage and identification probability in both experiments. This complex was therefore characterized in more detail (Table 1).

**Table 1:** Hey1 co-purified proteins identified by LC-MS/MS from whole cell (WC) and/or nuclear extracts (N) analyzed in the present study.

Identified Proteins	Description	Accession number	Unique Peptides		Coverage (%)	
			WC	N	WC	N
HEY1	Hairy/enhancer-of-split related with YRPW motif 1	117606332	18	15	56	47
HES1	Hairy and enhancer of split 1 isoform 1	114591154	6	7	25	26
PAM (MYCBP2)	Protein associated with myc	126116565	177	70	49	20
FBXO45	F-box protein 45	157743247	11	7	48	26
SKP1 (OCP-II protein)	S-phase kinase protein 1	114601679	9		60	

### FBXO45 interacts with Hey proteins, but not Hes1

**Fig 2:**
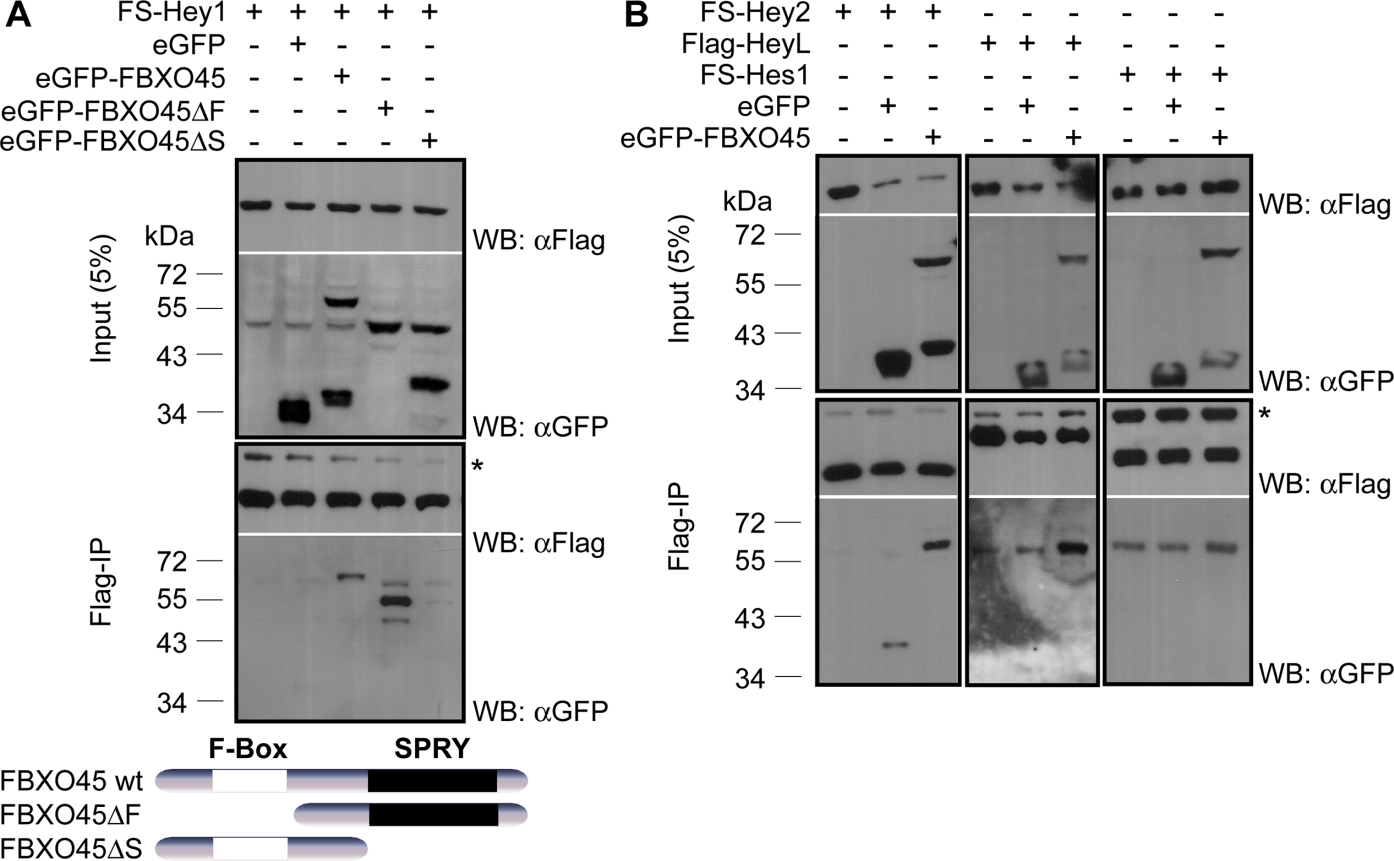
FBXO45 interaction with Hey family members. (A) Only the full-length and SPRY domain containing deletion mutant of FBXO45 co-precipitate with Hey1, while the F-box is not bound. Shorter presumed degradation products of eGFP-FBXO45 and eGFP-FBXO45ΔS visible in the input did not interact. FBXO45 deletions are depicted below. (B) Hey2 and HeyL, but not Hes1 interact with FBXO45. Doxycycline induced FS-Hey2,-Hes1 and Flag-HeyL cells were transfected with expression constructs for eGFP or eGFP-FBXO45 as indicated. IP was performed with an anti-Flag antibody. Lysates and precipitates were analyzed by Western blot with anti-Flag and anti-GFP antibodies. eGFP served as a negative control. The asterisk indicates IgG heavy chain.

The interaction between Hey1 and FBXO45 was verified by co-immunoprecipitation (co-IP) from induced 293tet-FS-Hey1 cells transfected with peGFP-FBXO45 (Fig 2A). To exclude artifacts due to tag sequences, co-IP was repeated with reciprocal HA- and Flag-tags with essentially the same results (S1 Fig). FBXO45 is characterized by two functional domains, a N-terminal F-box domain and a C-terminal SPRY domain [16]. Both domains are known to mediate protein-protein interactions [29, 30]. Analysis of FBXO45 deletion mutants revealed that the SPRY domain mediates the interaction with Hey1 (Fig 2A).

To test whether SPRY domains in general interact with Hey1 we performed IP experiments with the SPRY domain containing protein 3 (SPRYD3) that had also been detected in both purification experiments (S1 Table). However, co-IP experiments did not support a direct interaction between SPRYD3 and Hey1 (data not shown).

To further characterize the specificity of the Hey-FBXO45 interaction we tested cell lines expressing Flag- or FS-tagged Hey2, HeyL and Hes1 proteins in a similar manner. Hey2 and HeyL were able to co-precipitate FBXO45 (Fig 2B). In contrast, the closely related Hes1 protein did not interact with FBXO45 (Fig 2B) indicating a specificity for Hey proteins.

### HLH and Orange domains jointly bind FBXO45

**Fig 3:**
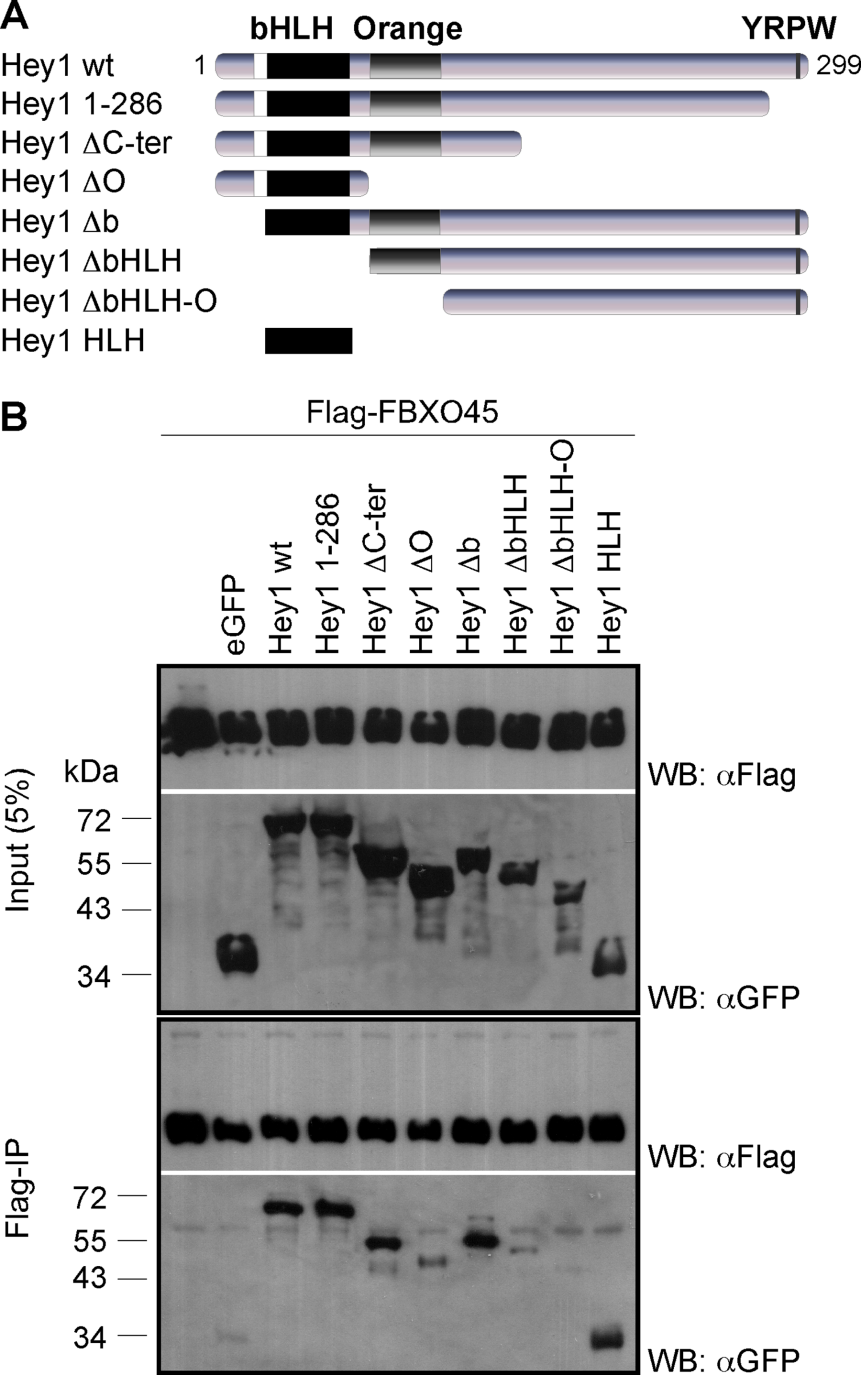
Hey1 HLH and Orange domains mediate FBXO45 interaction. (A) Schematic representation of eGFP-fused Hey1 deletion mutants. (B) Lysates from HEK293T cells co-transfected with Flag-FBXO45 and eGFP-Hey1 deletion mutants were immunoprecipitated with an anti-Flag antibody and tested by Western blot analysis as indicated.

The HLH domain of Hey1 mediates homo- and heterodimerization [7, 10] and the Orange domain has also been described to be involved in protein-protein interactions [31]. To determine the domain of Hey1 interacting with FBXO45, we performed co-IP experiments with Flag-FBXO45 and several Hey1 deletion mutants fused to eGFP (Fig 3A). Neither deletion of the Hey1 basic domain (Hey1 Δb) nor the successive truncation of the Hey1 C-terminus (Hey1 1–286; Hey1 ΔC-ter) abolished the interaction with FBXO45. In contrast, additional deletion of the HLH domain (Hey1 ΔbHLH) or the Orange domain (Hey1 ΔO) clearly weakend the ability to co-precipitate FBXO45. Only the combined deletion of the HLH and Orange (Hey1 ΔbHLH-O) domain completely abrogated the interaction with FBXO45. The Hey1 HLH domain alone was already able to bind FBXO45 (Fig 3B). Thus, the FBXO45 interaction appears to be jointly mediated by the HLH and the Orange domains without contributions from the C-terminal conserved domain.

### Hey1 draws FBXO45 into the nucleus

**Fig 4:**
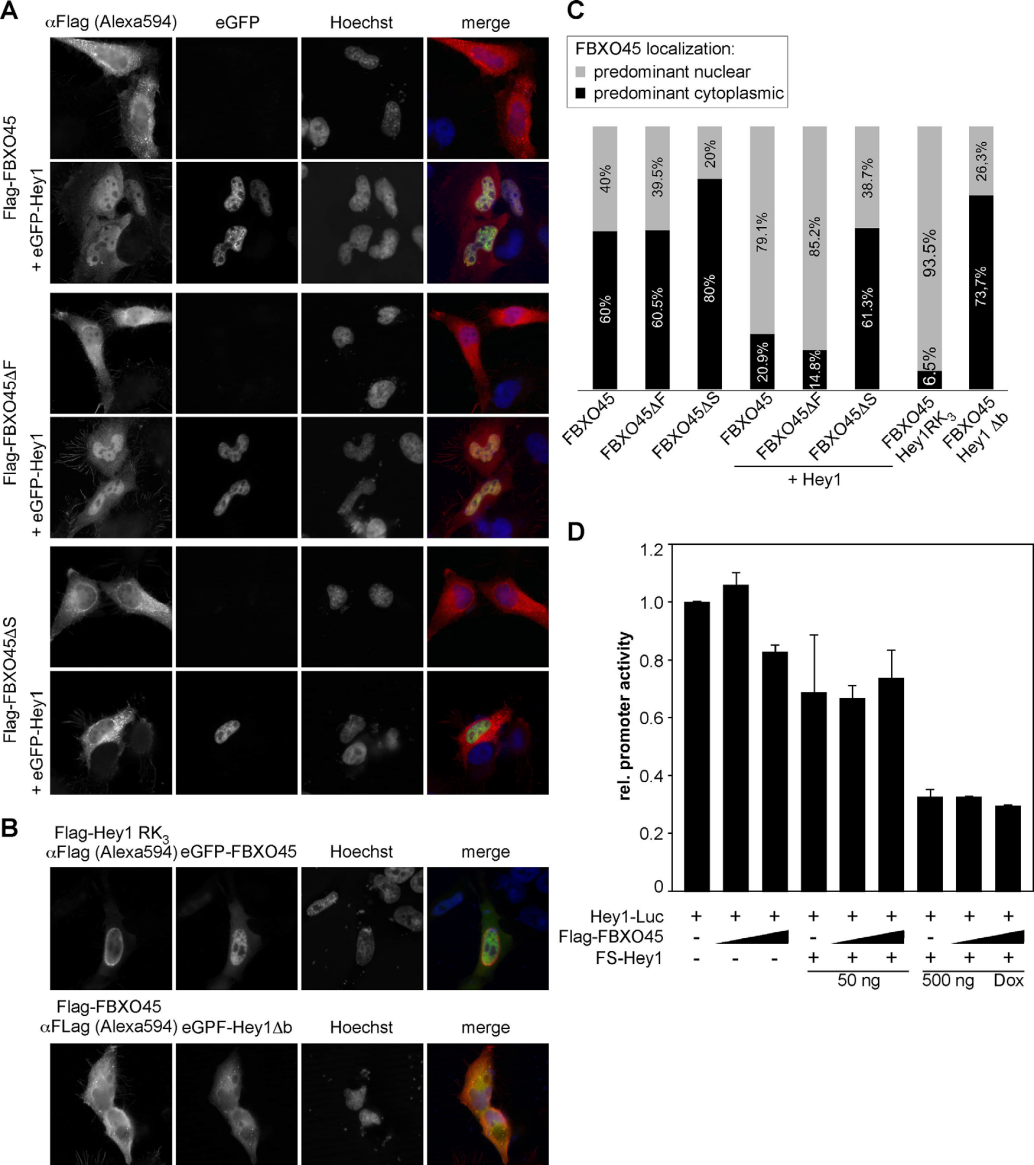
Co-expression of Hey1 strongly increases FBXO45 nuclear localization, but does not alter Hey repression capacity. (A) HeLa cells were transfected with FBXO45 expression vectors or its deletion mutants, either alone or together with peGFP-Hey1. Flag-tagged proteins were visualized with an anti-Flag antibody and an Alexa594 labeled secondary antibody. GFP fusion proteins were imaged directly and nuclei were stained with Hoechst33342 (B) The DNA-binding incompetent Hey1-RK_3_ variant and the Hey1Δb mutant that lacks the nuclear localization signal were expressed together with FBXO45 and analyzed as before. (C) Quantification of predominant cytoplasmic or nuclear FBXO45 staining. 80 cells were counted per preparation. (D) FBXO45 does not influence Hey1 transcriptional activity. 293tet-FS-Hey1 cells stably transfected with a Hey1-luc (2,9 kb) reporter construct were induced with doxycycline (50 ng/ml and 500 ng/ml for 72 h) to induce FS-Hey1 expression. After 48 h the cells were transfected with increasing amounts of Flag-FBXO45 (100 ng and 200 ng) and tested after additional 24 h. Mean values and standard deviations of triplicates are shown.

While Hey proteins typically reside in the nucleus, F-box proteins can be found in both, the cytoplasm as well as in the nucleus (reviewed in [32, 33]) and site preferences may even change, e.g. during the cell cycle [34]. We therefore investigated if the subcellular localization of FBXO45 may be altered by Hey1 co-expression. Full-length FBXO45, when expressed alone, shows a predominant cytoplasmic localization in HeLa cells. Upon co-expression of Hey1 a significant increase in nuclear located FBXO45 could be detected (Fig 4A). Nuclear colocalization of both proteins could be demonstrated by confocal microscopy (S2 Fig). A similar effect was observed using the FBXO45ΔF deletion mutant. In contrast, the predominant cytoplasmic localization of the FBXO45ΔS deletion mutant only slightly changed upon Hey1 co-expression, which confirms the lack of interaction seen in the co-precipitation experiments. A quantification of the data is shown in Fig 4C.

To evaluate whether DNA binding of Hey1 is needed to shift FBXO45 into the nuclear compartment, we used the Hey1-RK_3_ mutant [11] that lacks DNA binding capacity, but still exhibits nuclear localization. This mutant still interacts with FBXO45 (S3 Fig) and its expression leads to a clear shift of FBXO45 into the nucleus (Fig 4B and 4C). The Hey1Δb mutant protein that lacks the basic domain and its inherent nuclear localization signal locates to the cytoplasm and there is no nuclear accumulation of FBXO45, which clearly supports a Hey1-dependent subcellular localization. In HEK293T cells, FBXO45 showed essentially the same behavior (data not shown). Substitution of FS-Hey1 by mCherry-Hey2 led to the same increase in nuclear localization of FBXO45 (S4 Fig).

Hey1 represses target promoters—including its own—through largely unknown mechanisms. Luciferase reporter gene assays with a chromosomally integrated Hey1 promoter construct did not provide any evidence that FBXO45 might modulate Hey1 transcriptional repression capacity (Fig 4D). Similar results we observed in transiently transfected HEK293 cells (data not shown).

These results clearly show that Hey proteins enforce a nuclear localization of FBXO45. Furthermore, Hey1 DNA binding ability is not necessary for this effect.

### Hey1 interacts indirectly with PAM and SKP1

**Fig 5:**
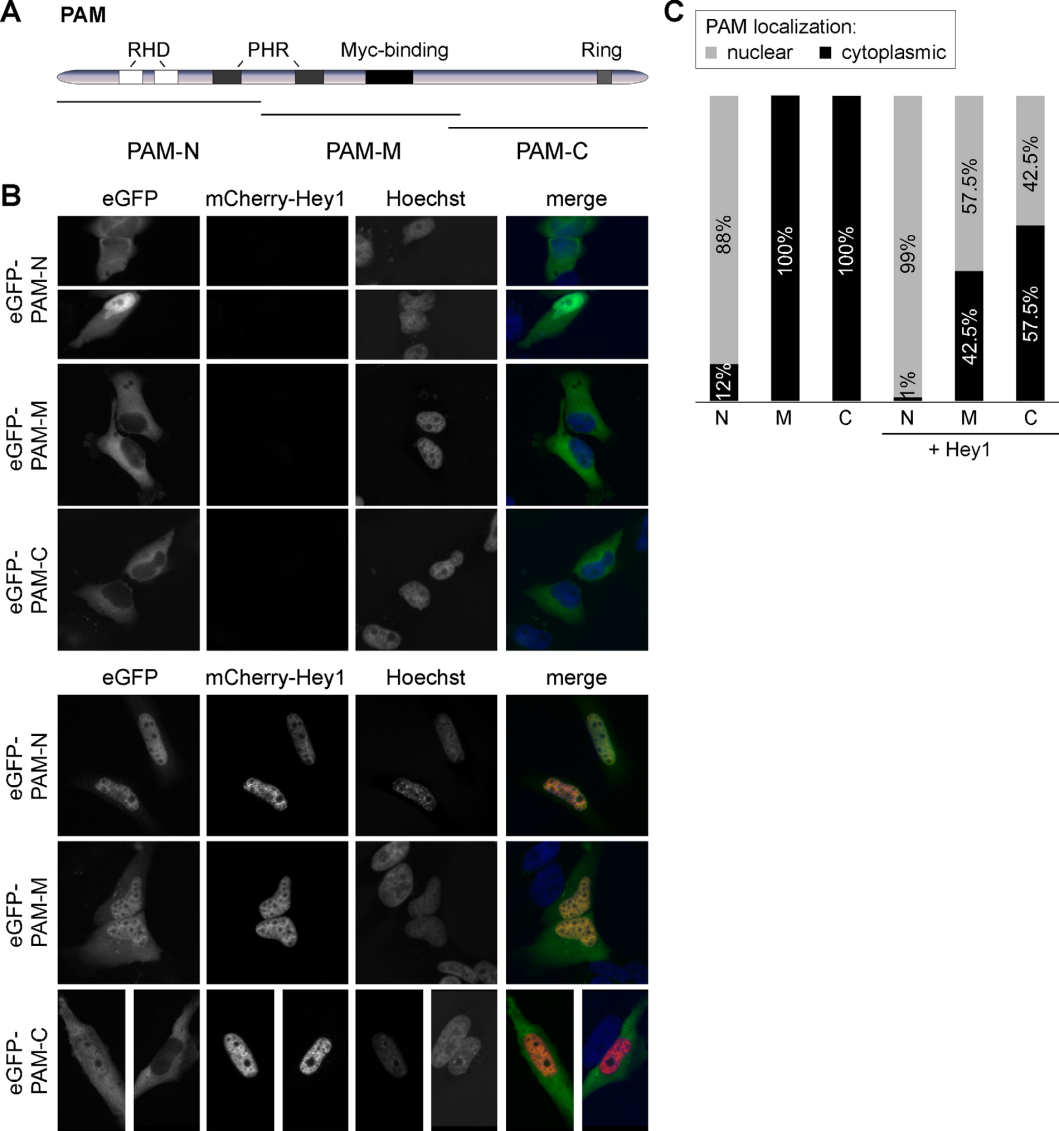
Hey1 redirects PAM fusion proteins. (A) Schematic representation of PAM-eGFP fusion constructs. RHD: RCC homology domains; PHR: PAM-Highwire-Rpm1 domain. (B) PAM fusion proteins translocate from the cytoplasm to the nucleus upon Hey1 co-expression. HeLa cells were transfected with eGFP-PAM deletion mutants alone or together with mCherry-Hey1. 24 h after transfection cells were fixed and nuclei were stained with Hoechst33342. (C) Quantification of cytoplasmic and nuclear PAM localization. 80 cells were counted per preparation.

**Fig 6:**
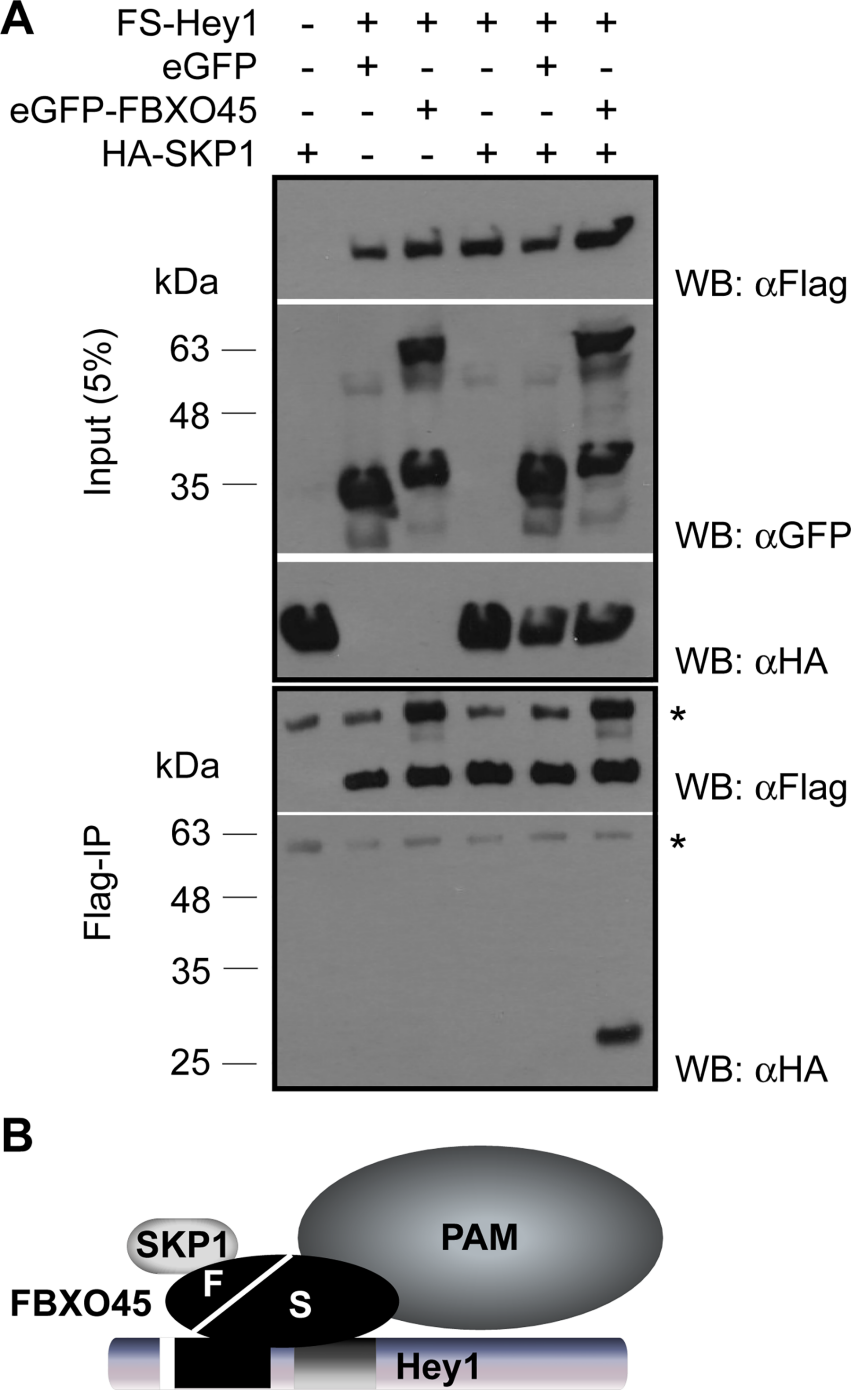
SKP1 indirectly co-precipitates with Hey1. (A) FS-Hey1 expressing cells were induced with doxycycline for 72 h. After 48 h the cells were transfected with expression constructs for eGFP, eGFP-FBXO45 or HA-SKP1, respectively. Lysates and immunoprecipitates were analyzed by Western blot with the appropriate anti-Flag, anti-GFP or anti-HA antibodies. The asterisk indicates for IgG heavy chain. (B) Scheme of Hey1, FBXO45, SKP1 and PAM complexes. FBXO45 functions as a linking part between PAM, SKP1 and Hey1. S: SPRY domain; F: F-Box.

PAM was the second highly ranked Hey1 interacting protein in our mass spectrometry analysis (Table 1). FBXO45 has been shown to form a complex with PAM and SKP1 through direct interaction [15, 16] and SKP1 was likewise co-purified with Hey1 in our analysis (Table 1). To validate the interaction of Hey1 with the very large 510 kDa PAM protein we generated several eGFP-fused PAM deletion mutants (Fig 5A) and tested these individually for Hey1 interaction in HeLa cells. The N-terminal part shows a predominant nuclear localization when expressed alone and this is not significantly affected by Hey1 co-expression. On the other hand, the middle and C-Terminal regions of PAM locate exclusively to the cytoplasm, but about half of the cells showed a clear nuclear accumulation in the presence of Hey1 (Fig 5B and 5C). Control experiments showed that FBXO45 itself interacts with the middle region of PAM, which is in agreement with earlier findings [16], and confirms the functionality of the proteins involved (S5 Fig). To find out whether the other E3-ligase component SKP1 directly interacts with Hey1 we performed co-IPs in the presence or absence of FBXO45. SKP1 was co-precipitated with Hey1 only if FBXO45 was included in the transfections demonstrating an indirect linkage (Fig 6A).

In Drosophila RPM-1 has recently been shown to interact with RAE-1 [35]. Since the human homolog RAE1 was also identified in our screen, there might be comparable interactions forming such a larger complex in human cells. However, we were unable to support a direct binding of RAE1 and Hey1 by co-immunoprecipitation (data not shown) suggesting that the interactions from the screening experiment may be indirect.

In summary, Hey1 seems to associate with the FBXO45/PAM/SKP1 E3-ligase complex, whereby FBXO45 functions as a linker protein between SKP1 and Hey1 as shown schematically in Fig 6B. Current data do not firmly distinguish between a direct or indirect interaction between Hey1 and PAM via FBXO45 at the moment.

### FBXO45 does not alter Hey1 stability or ubiquitination

**Fig 7:**
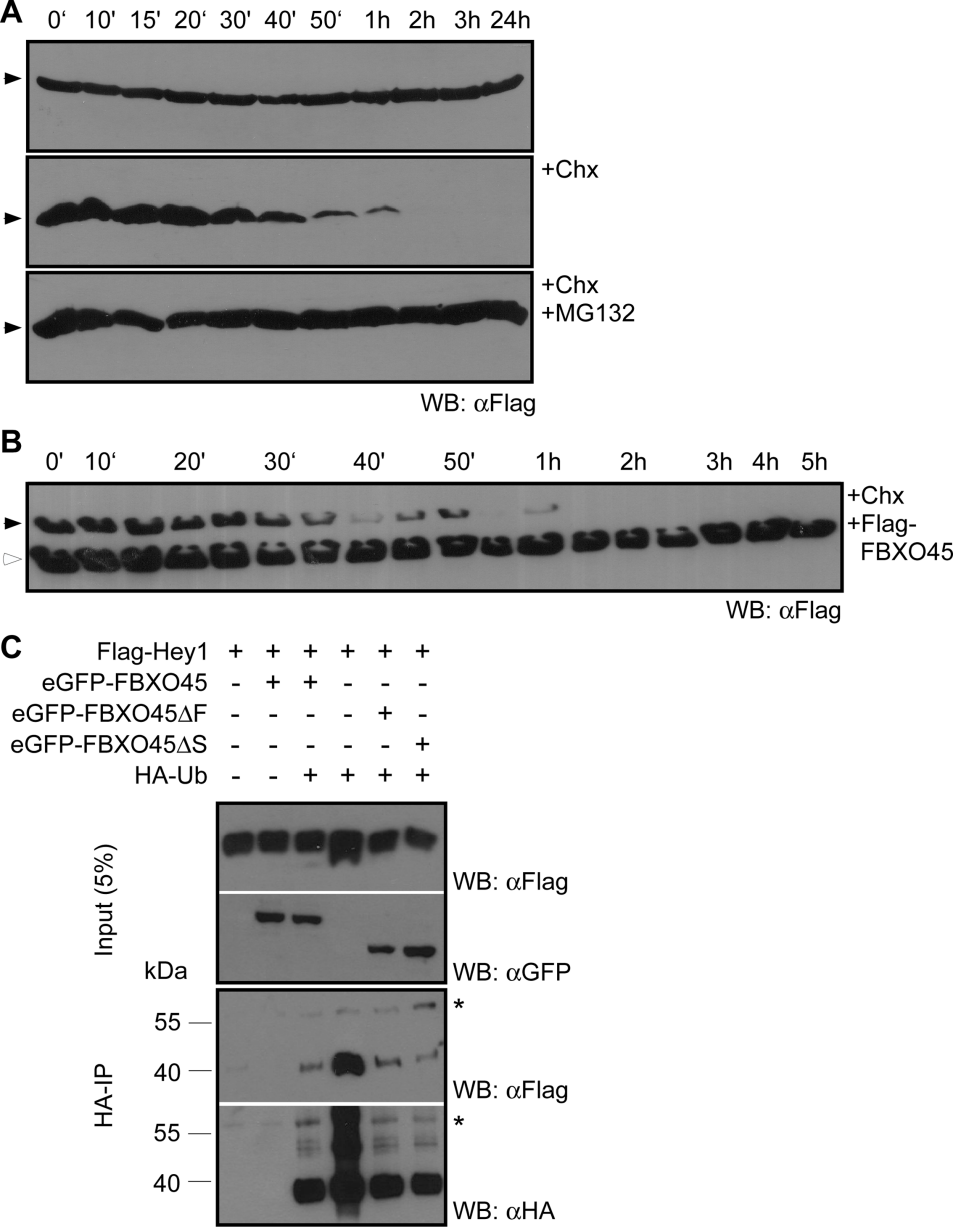
FBXO45 neither influences Hey1 stability nor its ubiquitination. (A) Hey1 expression was induced with doxycycline in 293tet-FS-Hey1 cells and tested by Western blot analysis at the time points indicated using an anti-Flag antibody (upper panel). Treatment with cycloheximide reveals a short half-life (middle panel), while addition of the proteasome inhibitor MG132 stabilizes Hey1 (lower panel). (B) Time course analysis upon co-expression of Flag-FBXO45 (white arrowhead) in the presence of cycloheximide. Hey1 is indicated by a black arrowhead. (C) FBXO45 co-expression does not induce Hey1 ubiquitination. HEK293T cells were co-transfected with Flag-Hey1, eGFP-FBXO45 fusion constructs and HA-Ubiquitin. HA immunoprecipitates were analyzed by Western blots with the antibodies indicated. The asterisk indicates the IgG heavy chain.

FBXO45 and its partner proteins are involved in protein polyubiquitination and FBXO45 has been shown to mediate ubiquitination of p73 leading to its proteasomal degradation [15, 18]. We therefore reasoned that FBXO45 might induce ubiquitination and degradation of Hey1. First, we evaluated the half-life time of Hey1 and assessed whether it is degraded by the proteasome. When translation was blocked in Dox-induced 293tet-FS-Hey1 cells with cycloheximide (Chx), a rapid loss of Hey1 signal was seen with an estimated half-life of 15 minutes (Fig 7A). Treatment with the proteasome inhibitor MG132 led to an extended Hey1 stability for ≥24 h, indicative of proteasomal degradation of Hey1 in untreated cells (Fig 7A). Unexpectedly, FBXO45 co-expression did not change Hey1 half-life time (Fig 7B).

We also tested for FBXO45 dependent ubiquitination of Hey1 *in vivo*. As shown in Fig 7C we could not detect (poly-)ubiquitinated Hey1 species upon expression of HA-tagged ubiquitin, independent of the presence or absence of FBXO45 or its deletion mutants. In control Western blots of the HA-IP using an anti-Flag antibody we detected only one distinct protein band occurring at approximately 40 kDa. This corresponds to the size of unmodified FS-tagged Hey1 that may be bound to ubiquitin-modified proteins, but is not modified itself. A repetition of the experiment in the presence of the inhibitor MG132 showed no differences (data not shown).

## Discussion

Hey proteins act as transcriptional repressors that form homo- or heterodimers. Besides their dimerization ability, they also recruit additional partners to form higher molecular weight complexes that may mediate their transcriptional effects. To better understand the mode of action of Hey proteins we utilized the tandem affinity approach combined with LC-MS/MS to isolate and identify endogenous interaction partners of Hey1. We could identify members of a known E3-ligase complex comprising FBXO45, SKP1 and PAM as novel Hey protein partners.

Hey synergistically uses its HLH and Orange domains to bind the SPRY domain of FBXO45. The SPRY domain has previously been reported to mediate the interaction of FBXO45 with PAM [16], but this does not appear to be mutually exclusive with Hey1 interactions. Our current data do not distinguish between a direct vs. indirect mode of Hey1-PAM interaction, however. On the other hand, the binding of SKP1 to FBXO45 is known to involve the F-box domain [36] and therefore FBXO45 is well suited to support the unhindered indirect interaction of Hey1 and SKP1, which was supported by experimental evidence. Our data are also in full agreement with recent analyses of Fbxo45 interactors in U87MG cells that found SPRY domain / PAM and F-box / Skp1 interactions [37]. We could not find the interaction of Fbxo45 with the extracellular domain of N-cadherin described in that publication, but this may be due to differences in subcellular localization.

### Hey1 controls localization of the FBXO45 complex

Co-expression of Hey1 leads to a strong nuclear accumulation of FBXO45. It is rather surprising that Hey1 is able to induce such a strong shift in subcellular localization of an E3-ligase complex that presumably serves many additional regulatory functions. A partial nuclear localization e.g. of FBXO45 is already observed without Hey1 overexpression, which may be due to endogenous Hey proteins although an additional Hey-independent process cannot be excluded. Analysis of FBXO45 using the nuclear localization signal (NLS) predictor [38] does not identify an intrinsic NLS. This suggests that FBXO45 needs a carrier for nuclear import other than the classical import system for which Hey1 would be an excellent candidate.

A similar mechanism appears to apply to transcription factors that regulate epithelial to mesenchymal transition (EMT). Zeb1/2, Snai1/2 and Twist1 have recently been described as targets of the Fbxo45 containing ubiquitin ligase complex in cancer cell lines [39]. Intriguingly, Zeb2 led to a nuclear translocation of Fbxo45 depending on a functional SPRY domain as documented for Hey1 in our case.

The cellular localization of PAM strongly depends on cell type and cell cycle phase [21, 40]. In HeLa cells Scholich et al. could show that PAM localizes exclusively to the cytoplasm during M phase, whereas during G1 phase PAM can be found in both compartments, the cytoplasm as well as the nucleus. Without forced Hey1 expression we found a strong nuclear signal of the N-terminal PAM deletion mutant whereas the other deletion mutants remain in the cytoplasm. This was surprising because only a C-terminal putative NLS of PAM is published [19, 41]. However, the NLS prediction software NLStradamus [38] predicts an additional N-terminal NLS (amino acids 53–88) that would explain the nuclear localization of the N-terminal PAM fusions (PAM-N), rendering the C-terminal NLS redundant. Most important, the nuclear translocation of PAM protein domains by co-expression of Hey1 indicates that the complete ubiquitin ligase complex seems to be redirected towards potentially novel targets.

### Hey1 is not ubiquitinated via Fbxo45

We could show that Hey1 is rapidly degraded by the proteasome, suggestive of Hey1 polyubiquitination. FBXO45 is known to mediate polyubiquitination of p73 leading to its proteasomal degradation [18]. The same is true for the EMT transcription factors described above as well as the pro-apoptotic tumor suppressor Par-4 [39, 42]. Furthermore, Gould and coworkers described Hey1 ubiquitination and degradation mediated by the viral E3-ligase RTA [43]. Nevertheless, we could not find evidence for Hey1 ubiquitination mediated by FBXO45. Co-precipitation of unmodified FS-Hey1 in Ubiquitin-IPs may rather indicate binding of Hey1 to an unidentified ubiquitinated protein. In summary, this suggests that Hey1 is not a regular substrate of FBXO45 and its associated E3-ligase complex, but might function as a mediator for ubiquitination of other proteins.

There are published data that F-box proteins influence other proteins independent of ubiquitination processes. The F-box protein MoKa functions as a co-activator for the Krüppel-like transcription factor KLF7. This interaction does not target KLF7 to the ubiquitination machinery [44]. According to our reporter gene assays (Fig 5E) we can exclude a similar effect of FBXO45 on Hey1 regarding transcriptional repression, ebut this may be different for other Hey1 target genes.

### *in vivo* relevance of the Hey1-FBXO45 interaction

The expression patterns of the genes and proteins involved appear to limit the sites relevant for the described Hey1 E3-ligase complex interaction *in vivo*. While PAM and FBXO45 are implicated in axon guidance and synapse formation [15, 16, 19], Hey proteins are involved in cell fate regulation and maintenance e.g. of neural precursors [33, 45]. In mice Pam and Fbxo45 expression appears highly restricted to the neural system. Both proteins show highest abundance in brain [15, 21, 46] and there seems to be a clear overlap with Hey1 in the hippocampus and the cerebellum [15] indicating that a Hey1-FBXO45 containing complex may act in neural development and/or maintenance, even if the Hey1 knock-out phenotype does not include striking neural defects.

The neural traits of the HEK293 cells used in the present approach may underlie the preferred isolation of neuron-specific potential interaction partners. In other cell types, there may be alternative combinations of SCF type complexes that can be bound and redirected by Hey1 proteins, but this may need proteomic analysis of Hey1 protein complexes from other source or comprehensive analysis of paralogous or sequence-related F-box proteins. On the other hand, FBXO45 has recently been implicated in the regulation of EMT and apoptosis in cancer cells [39, 42]. At the same time there is a newly discovered role of HEY proteins e.g. in breast and prostate cancer, which may point to additional places of functional overlap [47, 48]. Since HEY proteins are also known regulators of EMT [49], it will be interesting to decipher these networks.

Taken together, our results indicate a specific mechanism through which Hey1 redirects the unusual FBXO45 linked E3-ligase complex into the nuclear compartment. This may allow for novel regulatory mechanisms of Hey transcriptional activity beyond recruitment of co-repressor complexes.

## Supporting Information

S1 Fig Confirmation of Hey1/FBXO45 interaction using reciprocal tags. Flag-FBXO45 and HA-Hey1 were transfected into HEK293T cells either alone or together and immunoprecipitated with Flag or HA antibodies to visualize protein complexes.
(TIF)

S2 Fig Confocal microscopy confirms nuclear accumulation of FBXO45 upon Hey1 expression. (A) HeLa cells were transfected with eGFP-FBXO45 and mCherry-Hey1 or (B) eGFP-FBXO45 alone. 24 h after transfection the cells were fixed and stained with Hoechst33342 for confocal microscopy. Scale bars (11μm) are indicated.
(TIF)

S3 Fig The Hey1-RK_3_ mutant interacts with FBXO45. Lysates from HEK293T cells co-transfected with Flag-Hey1, Flag-Hey1-RK_3_, eGFP and eGFP-FBXO45 were tested by IP with an anti-Flag antibody. Lysates and precipitates were analyzed by Western blot with anti-Flag and anti-GFP antibodies. The asterisk indicates the IgG heavy chain.
(TIF)

S4 Fig Hey2 co-expression leads to increased FBXO45 nuclear localization. (A) HeLa cells were co-transfected with eGFP-FBXO45 and mCherry-Hey2. 24 h after transfection the cells were fixed and nuclei were stained with Hoechst33342. (B) Quantification of predominant cytoplasmic or nuclear FBXO45 staining. 80 cells were counted.
(TIF)

S5 Fig The deletion mutant PAM-M containing the Myc binding domain interacts with FBXO45. HEK293T cells were co-transfected with Flag-FBXO45 and eGFP-PAM deletion mutants. Lysates were taken for immunoprecipitation experiments using an anti-Flag antibody. Lysates and precipitates were analyzed by Western blot using anti-Flag or anti-GFP specific antibodies.
(TIF)

S1 Table Hey1 co-purified proteins identified by LC-MS/MS from whole cell (WC) and nuclear extracts (N). (DOCX)
