# Supplementary figures and images for: Hey bHLH Proteins Interact with a FBXO45 Containing SCF Ubiquitin Ligase Complex and Induce Its Translocation into the Nucleus
Source: PLoS One. 2015 Jun 12;10(6):e0130288. doi: 10.1371/journal.pone.0130288 (PMC4466309; doi:10.1371/journal.pone.0130288)

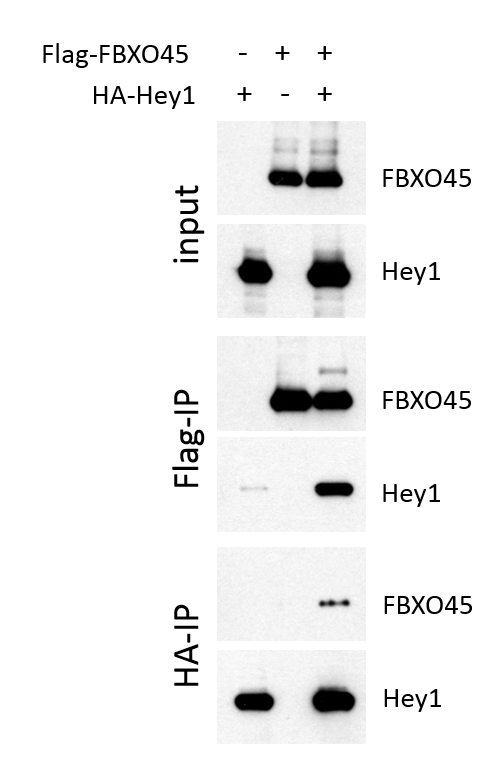

Supplement: S1 Fig — Flag-FBXO45 and HA-Hey1 were transfected into HEK293T cells either alone or together and immunoprecipitated with Flag or HA antibodies to visualize protein complexes. (TIF) [file pone.0130288.s001.tif]

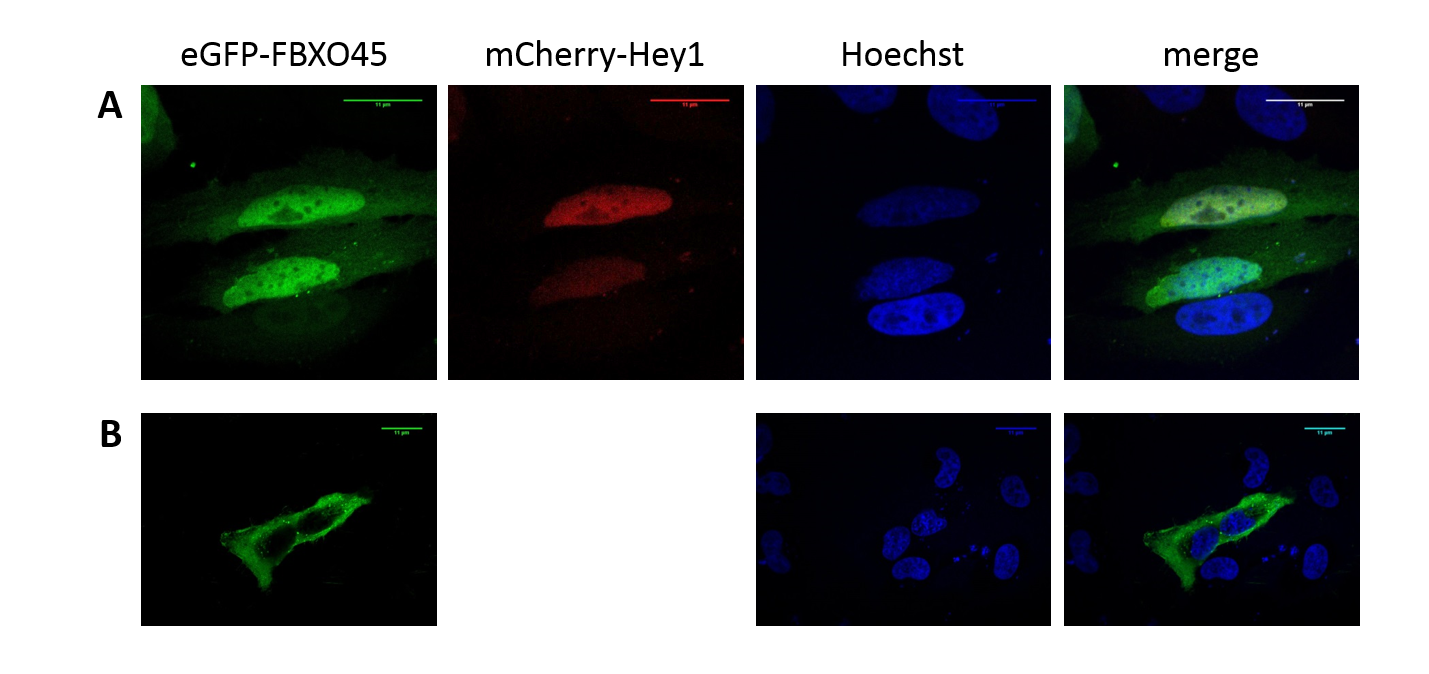

Supplement: S2 Fig — (A) HeLa cells were transfected with eGFP-FBXO45 and mCherry-Hey1 or (B) eGFP-FBXO45 alone. 24 h after transfection the cells were fixed and stained with Hoechst33342 for confocal microscopy. Scale bars (11μm) are indicated. (TIF) [file pone.0130288.s002.tif]

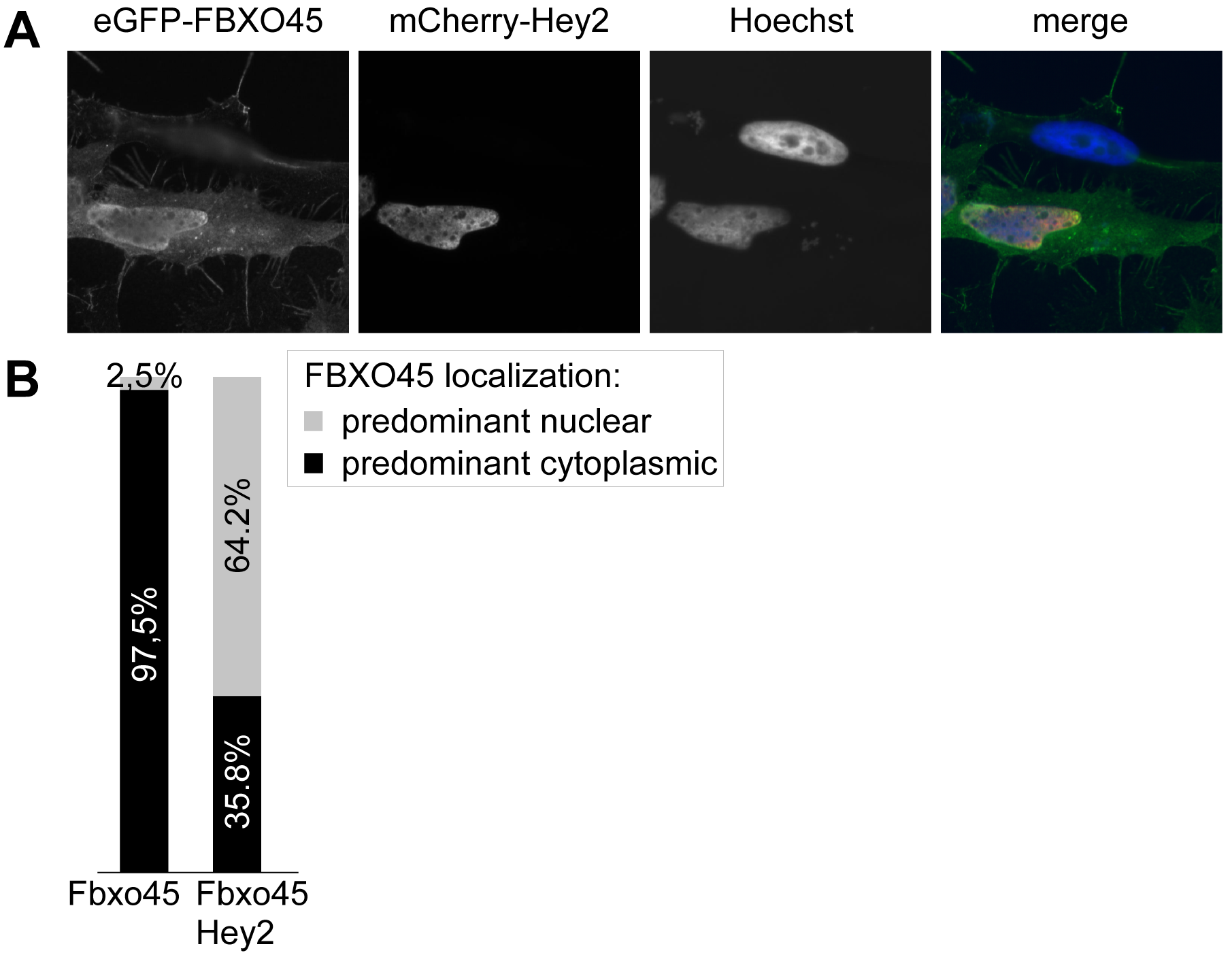

Supplement: S3 Fig — Lysates from HEK293T cells co-transfected with Flag-Hey1, Flag-Hey1-RK3, eGFP and eGFP-FBXO45 were tested by IP with an anti-Flag antibody. Lysates and precipitates were analyzed by Western blot with anti-Flag and anti-GFP antibodies. The asterisk indicates the IgG heavy chain. (TIF) [file pone.0130288.s003.tif]

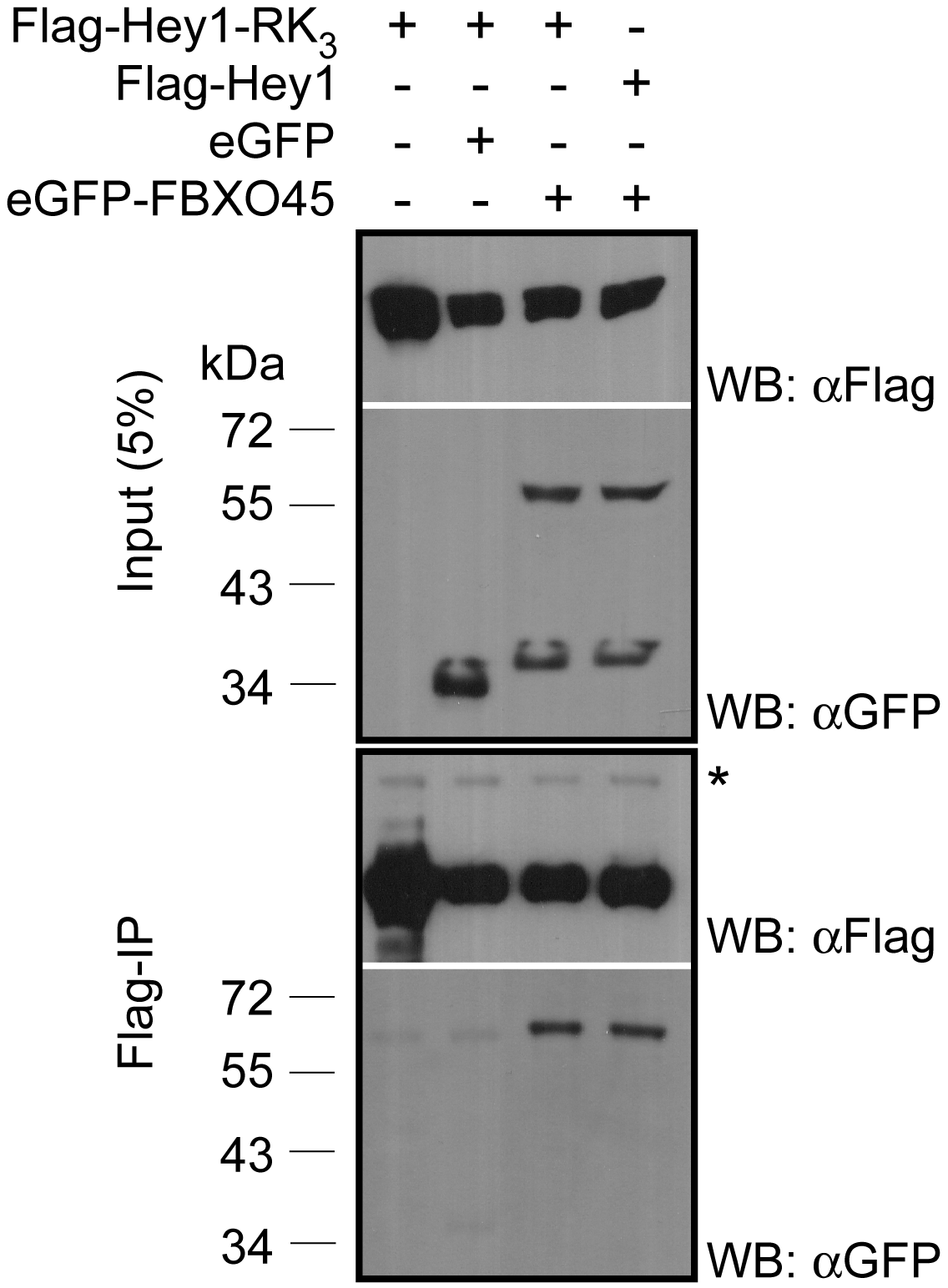

Supplement: S4 Fig — (A) HeLa cells were co-transfected with eGFP-FBXO45 and mCherry-Hey2. 24 h after transfection the cells were fixed and nuclei were stained with Hoechst33342. (B) Quantification of predominant cytoplasmic or nuclear FBXO45 staining. 80 cells were counted. (TIF) [file pone.0130288.s004.tif]

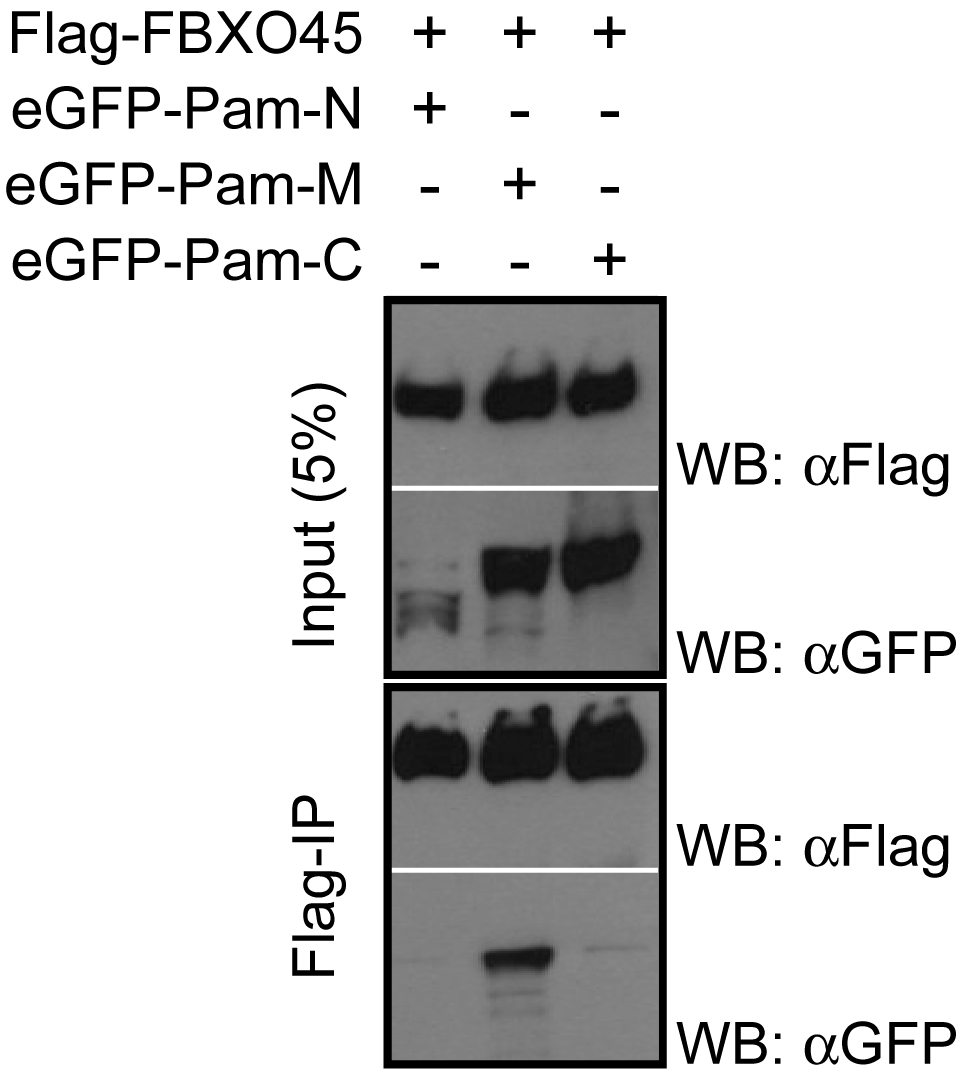

Supplement: S5 Fig — HEK293T cells were co-transfected with Flag-FBXO45 and eGFP-PAM deletion mutants. Lysates were taken for immunoprecipitation experiments using an anti-Flag antibody. Lysates and precipitates were analyzed by Western blot using anti-Flag or anti-GFP specific antibodies. (TIF) [file pone.0130288.s005.tif]
