# Supplementary material for: Hey bHLH Proteins Interact with a FBXO45 Containing SCF Ubiquitin Ligase Complex and Induce Its Translocation into the Nucleus
Source: PLoS One. 2015 Jun 12;10(6):e0130288. doi: 10.1371/journal.pone.0130288 (PMC4466309; doi:10.1371/journal.pone.0130288)
Supplement: S1 Table — (DOCX) [file pone.0130288.s006.docx]

| **Identified Proteins** | **Accession number** | **Unique Peptide** | | **Coverage (%)** | |
| --- | --- | --- | --- | --- | --- |
|  |  | WC | N | WC | N |
| CCT6A; Chaperonin containing TCP1, subunit 6A | 114613463 | 3 | 3 | 11 | 8,3 |
| CCT8; chaperonin containing TCP1, subunit 8 (theta) | 1136741 | 6 | 5 | 15 | 10 |
| FBXO45; F-box protein 45 | 157743247 | 11 | 7 | 48 | 26 |
| GCN1L1; GCN1 general control of amino-acid synthesis 1-like 1 isoform 3 | 114647251 | 7 | 13 | 3,4 | 6,3 |
| HDAC2; Histone deacetylase 2 | 114608973 | 2 | 3 | 5,3 | 7,4 |
| HES1; Hairy and enhancer of split 1 isoform 1 | 114591154 | 6 | 7 | 25 | 26 |
| HEY1; Hairy/enhancer-of-split related with YRPW motif 1 | 117606332 | 18 | 15 | 56 | 47 |
| HNRNPM; Heterogeneous nuclear ribonucleoprotein M | 119589327 | 2 | 20 | 4,1 | 32 |
| HSP70; Heat shock protein 70 | 126306139 | 1 | 2 | 21 | 18 |
| HSPD1; Mitochondrial heat shock 60kD protein 1 variant 1 | 189502784 | 13 | 14 | 34 | 33 |
| HSPH1; Heat shock 105kD | 114649455 | 12 | 4 | 14 | 5 |
| IMMT; inner membrane protein, mitochondrial | 114578614 | 21 | 2 | 33 | 3,8 |
| KHSRP; KH-type splicing regulatory protein (FUSE binding protein 2) | 114674909 | 4 | 3 | 8,9 | 5,2 |
| LGALS3; Lectin, galactoside-binding, soluble, 3 binding protein | 119609949 | 4 | 4 | 7,4 | 8,8 |
| MCM7; P1cdc47 | 1255617 | 6 | 4 | 12 | 7,6 |
| NUP188; nucleoporin 188kDa | 1136398 | 2 | 12 | 1,9 | 10 |
| PAM (MYCBP2); Protein associated with myc | 126116565 | 177 | 70 | 49 | 20 |
| PIP; Prolactin-induced protein | 116642259 | 2 | 2 | 27 | 27 |
| PRKDC; Protein kinase, DNA-activated, catalytic polypeptide | 119607088 | 58 | 69 | 19 | 21 |
| PYCR2; Pyrroline-5-carboxylate reductase family, member 2 isoform 6 | 114572916 | 8 | 13 | 26 | 41 |
| RAE1; RNA export 1 homolog | 4506399 | 15 | 7 | 46 | 26 |
| SPRYD3; SPRY domain-containing protein 3 | 14249554 | 10 | 4 | 36 | 10 |
| USP7; Ubiquitin specific protease 7 | 114660934 | 28 | 8 | 36 | 8,6 |
| USP9X; Ubiquitin specific peptidase 9 | 119579810 | 7 | 4 | 3,8 | 2,1 |
